# Supplementary material for: Addiction of mesenchymal phenotypes on the FGF/FGFR axis in oral squamous cell carcinoma cells
Source: PLoS One. 2019 Nov 4;14(11):e0217451. doi: 10.1371/journal.pone.0217451 (PMC6827898; doi:10.1371/journal.pone.0217451)
Supplement: S1 File — (DOC) [file pone.0217451.s001.doc]

**S1 File. Supporting Information**

**Materials and methods**

**Plasmid construction and luciferase Assays**

Human FGFR1c was obtained by RT-PCR using cDNAs of OSCC cells as a template, and was HA epitope-tagged at its C-terminus, followed by subcloning into pcDNA3.0 expression vector. Cells were seeded in duplicate in 24-well tissue culture plates, followed by transient transfection with various combinations of Activator protein 1 (AP-1) promoter-reporter constructs (AP-1 Luc)[32] and expression plasmids. Luciferase activity in cell lysates was determined with a dual luciferase reporter assay system (Promega) using a luminometer (AutoLumat LB953, EG&G Berthold, Natick, MA). Luciferase activity was normalized to sea-pansy luciferase activity of cotransfected phRL-TK plasmid (Promega) [6].
