# Supplementary figures and images for: Addiction of mesenchymal phenotypes on the FGF/FGFR axis in oral squamous cell carcinoma cells
Source: PLoS One. 2019 Nov 4;14(11):e0217451. doi: 10.1371/journal.pone.0217451 (PMC6827898; doi:10.1371/journal.pone.0217451)

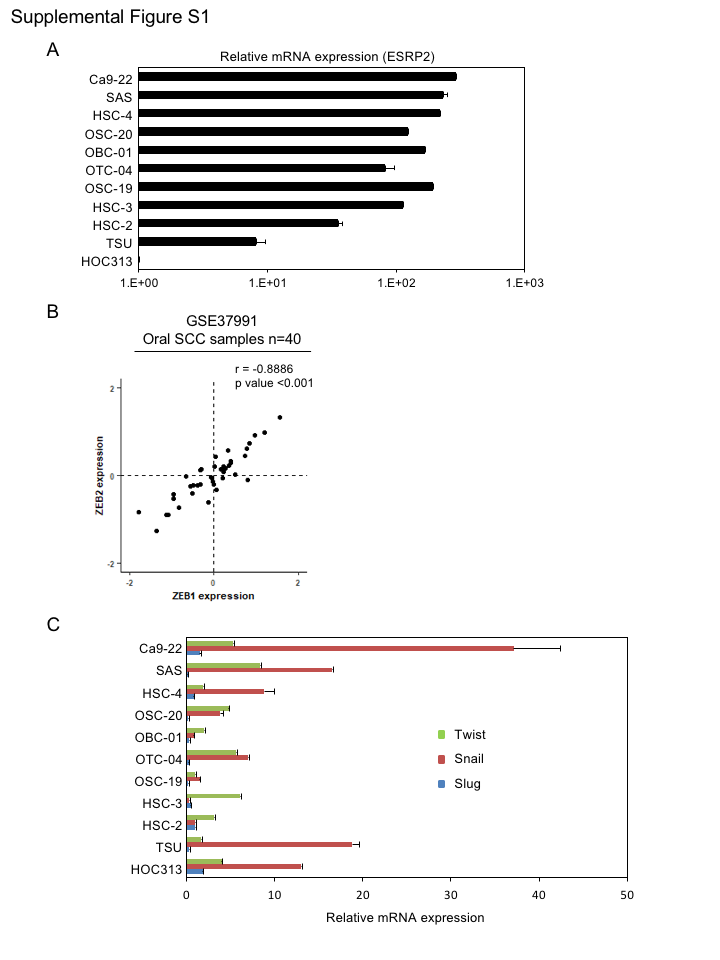

Supplement: S1 Fig — (A) ESRP2 mRNA level was determined by RT-qPCR in OSCC cells. Each value was normalized to the level of GAPDH mRNA in the same sample. The ratio of ESRP2 to GAPDH mRNAs in HOC313 cells was indicated as “1”. (B) Correlations between ZEB1 and ZEB2 mRNAs in oral cancer tissues from oral SCC patients in The Cancer Genome Atlas (TCGA) dataset were shown. TCGA is available from the website of The Cancer Genome Atlas program (National Cancer Institute). ZEB1 and ZEB2 mRNA expression in oral squamous cell carcinoma (SCC) patients were extracted from TCGA’s data portal (GSE37991). Statistical analysis revealed a positive correlation between the expression levels of ZEB1 and ZEB2 mRNA in 40 patients of oral SCC. (C) Expression level of Snail (SNAI1), Slug (SNAI2) and Twist mRNA was determined by RT-qPCR in OSCC cells. Each value was normalized to the level of GAPDH mRNA in the same sample. The ratio of Snail to GAPDH mRNAs and Slug to GAPDH mRNAs in HSC-2 cells was indicated as “1”. The ratio of Twist to GAPDH mRNAs in OSC-19 cells was indicated as “1”. Data are presented as means ± SD. (TIFF) [file pone.0217451.s002.tiff]

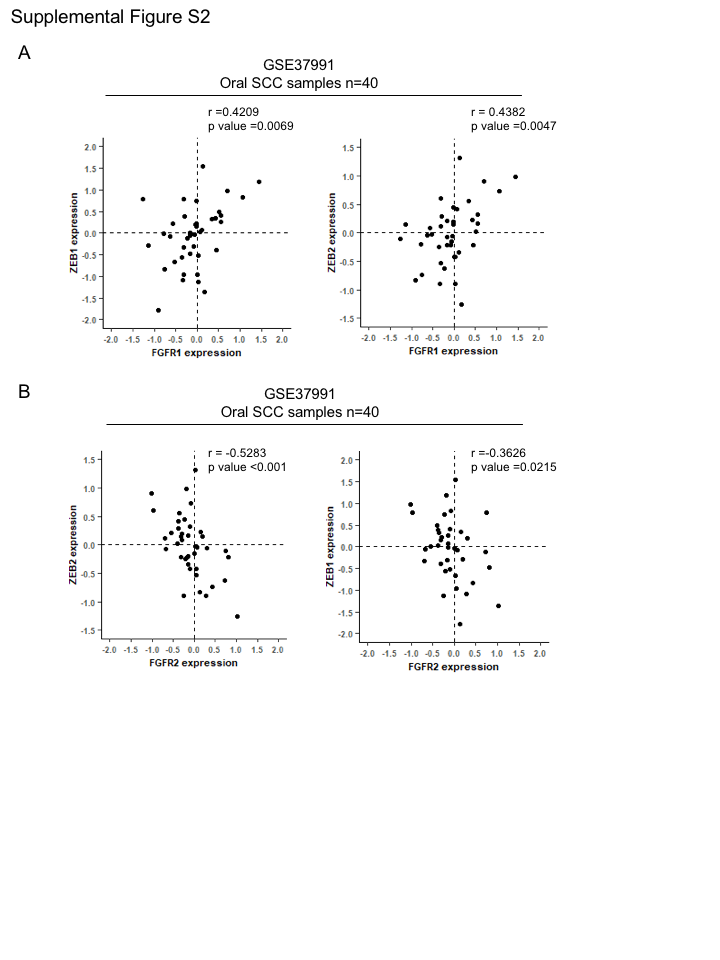

Supplement: S2 Fig — (A, B) Correlations between ZEB1/ZEB2 and FGFR1/FGFR2 in oral cancer tissues from oral SCC patients in TCGA dataset were shown. TCGA is available from the website of The Cancer Genome Atlas program (National Cancer Institute). ZEB1, ZEB2, FGFR1, and FGFR2 mRNA expression in oral squamous cell carcinoma (SCC) patients were extracted from TCGA’s data portal (GSE37991). Statistical analysis revealed a positive correlation between the expression levels of FGFR1 and ZEB1 (left) or ZEB2 (right) mRNA (A), and a negative correlation between the expression levels of FGFR2 and ZEB1 (left) or ZEB2 (right) mRNA (B) in 40 patients of oral SCC. (TIFF) [file pone.0217451.s003.tiff]

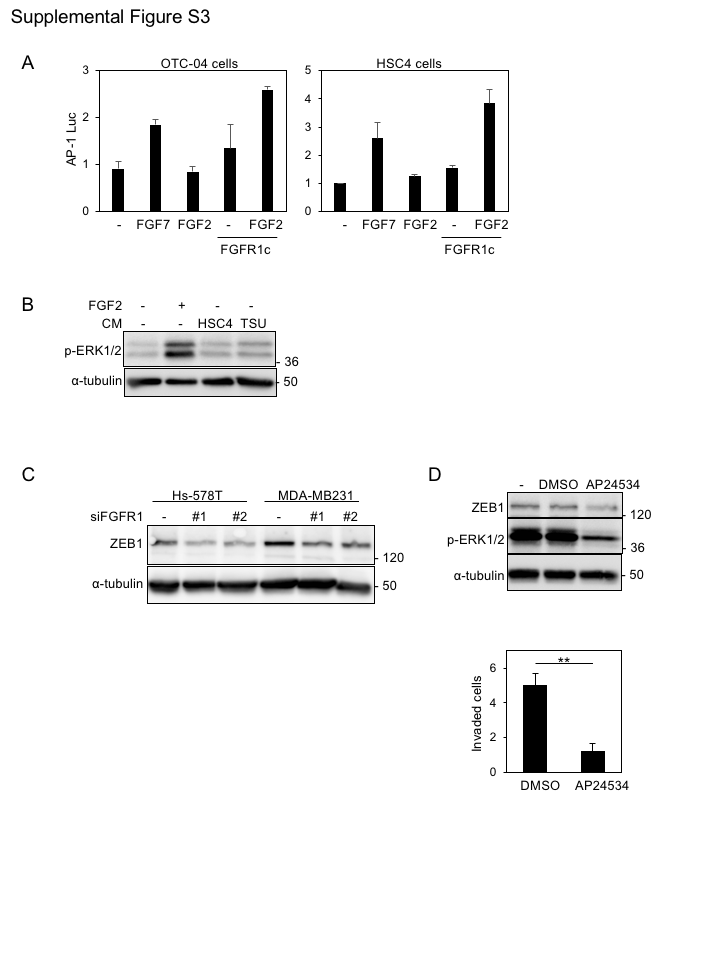

Supplement: S3 Fig — (A) OTC-04 and HSC4 cells were cotransfected with AP-1 promoter-reporter construct (Ap-1 Luc.) in combination with FGFR1c-expression plasmids. At 24 h after transfection, the cells were stimulated with either FGF-7 or FGF-2. Twelve h later, the cells were harvested and assayed for luciferase activity. (B) After NMuMG cells were pretreated with TGF-β, the cells were further incubated in the conditioned medium (CM) from either HSC4 or TSU cells. FGF2 was used as a positive control. (C) The basal-like subtype of breast cancer cells, Hs-578T and MDA-MB231 cells, are known to express FGFR1(IIIc) [6]. ZEB1 levels were also determined in these cells transfected with siFGFR1. (D) TSU cells treated with 10 μM AP24534 in the absence of FBS were subjected to immunoblotting using the indicated antibodies (top panels) and to Boyden chamber assays (bottom panel). α-tubulin was used as a loading control (B, C and D). Each value represents the mean ± SD of triplicate determinations from a representative experiment. Similar results were obtained from at least three independent experiments. p values were determined by Student’s t-test. **p < 0.01. (TIFF) [file pone.0217451.s004.tiff]
